# Methodological challenges in following up patients of a hospital child protection team: is there a recruitment bias?

**DOI:** 10.1186/1753-2000-4-27

**Published:** 2010-11-04

**Authors:** Andreas Jud, Ulrich Lips, Markus A Landolt

**Affiliations:** 1 University Children's Hospital Zurich, Steinwiesstrasse 75, 8032 Zürich, Switzerland

## Abstract

**Background:**

The aims of this study are to describe the methodological challenges in recruiting a follow-up sample of children referred to an interdisciplinary hospital child protection team (CPT) and to compare participating versus non-participating groups on several demographic variables and maltreatment characteristics.

**Methods:**

Of the 319 in- and outpatients referred to the CPT at University Children's Hospital Zurich from 2005–2006 a sample of 180 children was drawn to contact for a follow-up. The children and their parents were asked to participate in a face-to-face interview at the hospital; in 42 cases the children and parents consented to do so. Alternatively, the parents could take part in a telephone interview (n = 39). Non-participation resulted because no contact or adequate communication in German, French, or English could be established (n = 49) or because the parents or children refused to participate (n = 50).

**Results:**

Participants and non-participants did not differ significantly in mean child age at follow-up, gender, family status, place of residence, certainty and type of maltreatment, and type of perpetrator. However, the child's nationality had a significant impact: Percentages of foreign nationals were higher in the fully participating group (45%; n = 19) and the non-contactable group (53%; n = 26) and significantly lower in the refusal (26%; n = 10) and the telephone interview group (18%; n = 9). Although a high percentage of families had moved in the few years since the CPT intervention (32%; n = 57), the percentage of moves was not significantly higher in non-participants compared to participants.

**Conclusions:**

Further research is needed to support these results in different national backgrounds and to test for biases in variables not included – especially socioeconomic status. This includes gathering more detailed information on non-participants, while respecting ethical boundaries. Overall, the fact that only child's nationality was unevenly distributed between participants and non-participants is encouraging.

## Background

In many countries, multidisciplinary team approaches to the diagnosis and treatment management of child maltreatment have been established and are now commonly used. However, only few methodologically sound and recently published papers reported data on child protection team (CPT) cases in hospitals [cf. [1]]. Empirical data on the intervention outcome of hospital CPTs is even scarcer [2-6]. Most of the few studies analyzed outcome using patient records or interviews with professionals who had subsequently supported the children or their families [2-5]. Only one study [6] followed up the maltreated children and their families directly; of the 187 children that met the study's inclusion criteria, 84 (45%) participated. Lynch et al. concluded that the most dysfunctional families were the least likely to participate in their study. However, of the non-participants, 25% declined to participate, and 75% were not invited to participate, because the social workers expected them to decline. On what basis the social workers made their decision was not reported. In response to that article, Feehan et al. [7] concluded that the evidence presented did not justify labeling these families dysfunctional, which makes the results difficult to interpret. As the results of maltreatment research may be biased by differences in participants, there is a need for analyses of participant characteristics.

Some years ago, Ammerman [8] addressed the lack of empirical data on participation in maltreatment research and discussed major challenges in subject recruitment and retention: Parents are likely to decline participation in research on child protection, because the studies often ask intrusive questions and deal with sensitive and private family matters. Parents may fear – subjectively reasoned or not – that there will be an intervention, an invasion of privacy. This may be especially true for families who have already had contact with a CPT. Refusal to participate in an intrusive study may be associated with characteristics of the maltreatment situation. Participation is probably less likely if the perpetrator is part of the family. Further, participation may be correlated with certainty and type of maltreatment. Empirical data regarding these participation barriers in maltreatment research are still lacking today.

Of course, people turn down participation in research studies for other reasons [8]. They may have neither time nor interest; they may lead especially chaotic and disorganized lives and be unable to make arrangements to visit a clinic – a reason which may often be found in maltreating families. Reviews of risk factors in child maltreatment [e.g., [9]] identified variables that are possibly connected with difficulties in participant recruitment: Maltreating families tend to move frequently and often do not have a telephone (and mobile phone numbers are not available). Time-related and logistic barriers to participation identified in other contexts [10,11] are likely to be found in families with maltreated children. Restricted time schedules in school age children, logistic demands of single parenthood, large distances, and difficulties in transportation may reduce participation in various study populations. Further, in foreign nationals inadequate understanding of a written and/or spoken language may be a further barrier to participation.

### Aims

The aim of this study was to gather information on groups participating and non-participating in an interview and to assess the role of characteristics of the maltreatment situation and sociodemographic variables in predicting non-participation of former patients of the CPT at University Children's Hospital Zurich.

As empirical and methodological knowledge on study participation in child maltreatment outcome research is quite scarce, the hypotheses to be tested have to remain on an exploratory level. First, we expected variables representing poor reachability/contactability (moves, foreign nationality) or variables associated with time-related and logistic barriers (school age of child, single parenthood, large distances) to be overrepresented in non-participating families. Second, we assumed that maltreatment characteristics associated with high intrusive quality (substantiated maltreatment, sexual abuse, intrafamilial perpetrator) are more common in non-participants.

## Methods

### Sample

**Figure 1:**
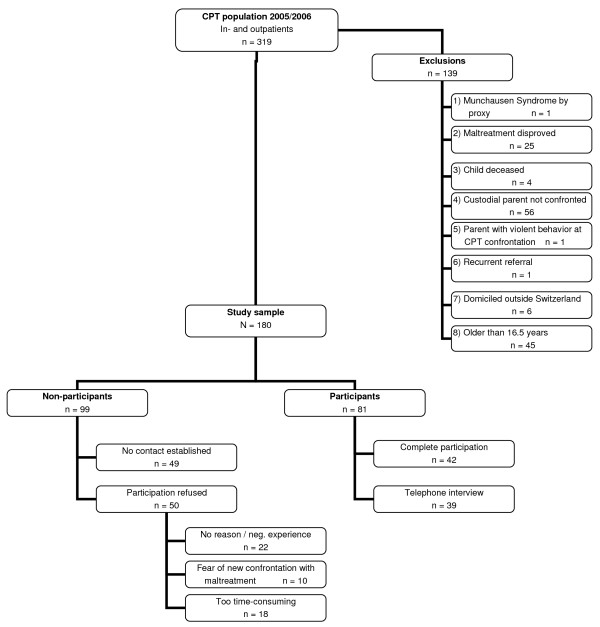
**Path to study sample with participating and non-participating children**. As certain children could have been excluded for several reasons, numbers per reason are listed according to their rank in excluding.

In the years 2005 and 2006 the CPT at University Children's Hospital Zurich visited 319 children as in- or outpatients; 139 children were excluded from the sample for different reasons such as Munchausen Syndrome by proxy (MSBP), or because the maltreatment had been disproved, the child was over the age of 16.5 years at the time of the follow-up contact (see Figure 1). A further category of exclusion comprised cases of custodial parents who had not been confronted with the fact that the CPT had discussed suspected maltreatment of their child, because no further child protection interventions were deemed necessary. The final sample of 180 children was drawn to contact for a follow-up interview, with the intention to analyze developmental outcomes of maltreated children in a variety of psychosocial and biological domains. The results on the developmental outcomes of participants will be reported elsewhere.

Eligible children and their parents were asked to participate in a face-to-face interview at University Children's Hospital Zurich; in 42 cases the children and parents consented to do so. Alternatively, the parents could take part in a telephone interview (n = 39). Non-participation resulted because no contact or adequate communication in German, French, or English could be established (n = 49) or because the parents or children refused to participate (n = 50). Demographic variables and characteristics of the maltreatment situation are described below in the results section. The research design was approved by the local ethics committee.

### Measures

Data collected at the initial referral to the CPT were used to analyze characteristics of non-participation, as these data were available for both participants and non-participants. Demographic data were available on the child's gender, age at follow-up, nationality, place of residence, moves, and family status. Nationality was dichotomized, with the child categorized as either Swiss or foreign national. As few patients resided outside the canton of Zurich and patients domiciled in foreign countries were excluded, the place of residence was dichotomized into residing in the city of Zurich and residing elsewhere. Family status was divided into three categories: families with two caregivers, single parents, and children placed externally.

Besides demographic variables, characteristics of the maltreatment situation were included in the analyses. Child maltreatment was categorized as physical, sexual, or psychological maltreatment, or neglect (for definitions see Table 1). The certainty of maltreatment was differentiated into substantiated or indicated. Relying on broadly accepted criteria [12], the maltreatment of a child was categorized as substantiated if physical or psychological symptoms were most likely explained by maltreatment or if the child disclosed the maltreatment to medical professionals. If maltreatment could be neither substantiated nor dismissed, it was judged to be indicated. The CPT coded one main type of maltreatment per child. Cases where children were suspected to suffer from multiple types of maltreatment were coded by the substantiated maltreatment type. If several categories were substantiated, physical or sexual maltreatment was coded instead of psychological maltreatment or neglect. Since the categories sexual (35%; n = 63) and physical maltreatment (31%; n = 56) were by far the most common types of maltreatment in our sample, the remaining categories with small numbers of cases were merged for further analyses. Perpetrators were categorized as intrafamilial or extrafamilial.

**Table 1:** Definitions of maltreatment types ^1 ^used by the CPT at University Children's' Hospital Zurich

*Type of maltreatment*	*Definition*
**Physical maltreatment**	Intentional use of physical force against a child that results in, or has the potential to result in, physical injury.
**Psychological maltreatment**	Intentional caregiver behavior that conveys to a child that he/she is worthless, flawed, unloved, unwanted, endangered, or of value only in meeting another's needs.
**Neglect**	Failure by the caregiver to provide basic physical and psychological needs and failure by the caregiver to ensure a child's safety within and outside the home given the child's emotional and developmental needs.
**Sexual maltreatment**	Any completed or attempted sexual act, sexual contact with, or exploitation of a child by a caregiver. Non-contact sexual maltreatment can include acts that expose a child to sexual activity, filming of a child in a sexual manner, sexual harassment, or prostitution of a child.

*Note. *^1 ^Extended versions of these definitions have been reported elsewhere [1].

To account for a possible informant bias, it was coded whether the primary contact for participants and contactable non-participants had been the mother, the father, or some other person (e.g., a legal guardian, an older sibling, or the index adolescent). Additionally, reasons for non-participation were asked on the telephone as an open-ended question; the coded results are described below.

### Procedure

The sampled children and their custodial parents were first sent an information letter and a written informed consent form. If the informed consent was not sent back within two weeks, the first author attempted to contact the family by telephone and using a standardized script. After five unsuccessful calls on different days of the week and at different times of the day, the family was sent a written reminder. If the reminder and subsequent telephone calls still led to no contact, the child was categorized as non-contactable. If a letter was returned because of an invalid address, the child's new address was searched for via telephone directories or registration offices. If a parent was reached by telephone but did not consent to participate fully, he/she was asked to answer a few questions on child behavior on the telephone.

### Statistical and descriptive analyses

Distributions of categorical variables in participating and non-participating groups were analyzed using chi-square tests and differences in age means using analysis of variance (ANOVA). The child's gender was analyzed to control for a possible bias in distribution. All statistical analyses were conducted using the software Stata 10 [13]. The statistical analyses are complemented by a qualitative description of difficulties in data collection.

## Results

### Characteristics of participating groups compared to non-participants

Table 2 presents frequencies or mean values for demographic variables in participating versus non-participating groups; Table 3 shows frequencies for maltreatment characteristics. There was a significant difference in distribution when looking at the child's nationality. Percentages of foreign nationals were high in the fully participating group (45%; n = 19) and the non-contactable group (53%; n = 26) and significantly lower in the refusal (18%; n = 9) and telephone interview group (26%; n = 10). More than half of the caregivers of children placed out-of-home refused participation. However, because the number of children placed out-of-home was very small (n = 12), this category was excluded from the comparison of family status, which did not reach statistical significance. A total of 57 former patients (32%) had moved since the CPT intervention. Their rate was not only high in the non-participating groups but also in the participating groups, with a percentage of 36% (n = 15) in complete participants; the difference between the groups was therefore not significant. None of the other demographic variables tested on their interaction with participation had an uneven distribution or were connected with a significantly higher or lower probability for one of the groups (Table 2). Additionally, neither the characteristics of the maltreatment situation (Table 3) nor the person of primary contact (Table 4) was associated with an uneven distribution in participating and non-participating groups.

**Table 2:** Frequencies or mean values for demographic variables in participating and non-participating groups

*Variable*	*Complete participation*	*Telephone interview*	*Refusal*	*No contact*	***χ***^***2***^***test or ANOVA***	
	*(n = 42)*	*(n = 39)*	*(n = 50)*	*(n = 49)*	***χ***^***2 ***^***(df) or F (df)***	*p*
**Gender (%)**						
Female	24 (57)	19 (49)	25 (50)	30 (61)	1.95 (3)	0.584
Male	18 (43)	20 (51)	25 (50)	19 (39)		
**Age at follow-up (SD)**	8.4 (3.8)	9.9 (3.6)	8.0 (4.4)	8.5 (4.4)	1.80 (3)	0.149
**Citizenship (%)**						
Swiss	23 (55)	29 (74)	41 (82)	23 (47)	16.67 (3)	0.001***
Foreign nationality	19 (45)	10 (26)	9 (18)	26 (53)		
**Family status (%)**						
Two caregivers	26 (65)	22 (59)	25 (53)	27 (56)	0.46 (3)^1^	0.928
Single caregiver	13 (32)	15 (41)	15 (32)	17 (35)		
Out-of-home placement^1^	1 (3)	0 (0)	7 (15)	4 (8)		
**Place of residence (%)**						
City of Zurich	15 (36)	12 (31)	14 (28)	25 (51)	6.57 (3)	0.087
Outside of city of Zurich	27 (64)	27 (69)	36 (72)	24 (49)		
**Moves (%)**						
Has not moved	27 (64)	29 (74)	36 (72)	31 (63)	1.86 (3)	0.601
Moved	15 (36)	10 (26)	14 (28)	18 (37)		

*Note*. Percentages are added in columns; ^1 ^the out-of-home-placement category was excluded from χ^2^test, as too many cell counts were below 5; ***p < .001.

**Table 3:** Frequencies for maltreatment characteristics in participating and non-participating groups

*Variable*	*Complete participation*	*Telephone interview*	*Refusal*	*No contact*	***χ***^***2***^***test***	
	*(n = 42)*	*(n = 39)*	*(n = 50)*	*(n = 49)*	*χ (df)*	*p*
**Type of maltreatment (%)**						
Sexual abuse	15 (36)	18 (46)	14 (28)	16 (33)	6.76 (6)	0.344
Physical maltreatment	11 (26)	11 (28)	21 (42)	13 (27)		
Other maltreatment	16 (38)	10 (26)	15 (30)	20 (41)		
**Certainty (%)**						
Substantiated	32 (76)	33 (85)	38 (76)	39 (80)	1.22 (3)	0.749
Indicated	10 (23)	6 (15)	12 (24)	10 (20)		
**Perpetrator (%)**						
Intrafamilial	26 (62)	25 (64)	35 (70)	35 (71)	1.28 (3)	0.734
**Extrafamilial**	16 (38)	14 (36)	15 (30)	14 (29)		

*Note*. Percentages are added in columns.

**Table 4:** Frequencies for primary contact in participating and non-participating groups

*Variable*	*Complete participation*	*Telephone interview*	*Refusal*	***χ***^***2***^***test***	
	*(n = 42)*	*(n = 39)*	*(n = 50)*	***χ***^***2 ***^***(df)***	*p*
**Primary contact (%)**					
Mother	34 (81)	33 (85)	30 (60)	1.60 (2)	0.450
Father	4 (10)	6 (15)	8 (16)		
Other person^1^	4 (10)	0 (0)	12 (24)		

*Note*. Percentages are added in columns; ^1 ^the "other person" category was excluded from χ^2^test, as too many cell counts were below 5.

### Reasons for non-participation and qualitative description of difficulties in data collection

Of the 50 children and parents refusing participation, 18 stated that participation was too time-consuming; among single parents refusing to participate, three-fifths (59%; n = 10) mentioned this reason. Ten parents or children did not want to be confronted again with the maltreatment and the events associated with it. A final 22 parents or children did not mention any reason for non-participation, a few of them ending the call as soon as they heard the words "University Children's Hospital Zurich." Many others responded to the call aggressively at first. Caregivers who participated in the telephone interview sometimes showed ambivalent behavior. They answered the call aggressively at first but then started to speak quite open-heartedly after the initial phase.

No contact could be established with 49 former patients. For some of the former patients telephone or mobile phone numbers were not available or (currently) out of order, and letters were not answered. Others answered neither telephone calls nor letters; mobile phone calls were sometimes refused. Yet others had moved out of Switzerland or had given an address at which they had never lived, and therefore no new contact could be searched and established. Finally, some parents answered the call but were not able to answer in German, French, or English and were not able to understand the meaning of the letter or the call.

## Discussion

Because difficulties in recruitment of participants for studies on child maltreatment may lead to biased samples, we compared participating versus non-participating groups with regard to several demographic variables and maltreatment characteristics. However, the only variable found to be associated with an uneven distribution in participating compared to non-participating groups was the child's nationality. The percentage of children with a foreign nationality was highest in the group where no adequate contact had been established and second highest in the fully participating group; the percentages of children with a foreign nationality were lower in the telephone interview and refusal groups.

Studies comparing the participation rate of hospital CPT patients at follow-up are lacking, with exception of the study by Lynch et al. [6]. But interpretation of the results of the Lynch et al. study is difficult because of impreciseness in defining participation. Based on an exploratory assumption, we therefore expected to find variables representing poor reachability or contactability to be overrepresented in non-participating groups. The significantly higher percentage of foreign nationals in the non-contactable group is not surprising given the fact that this group includes cases where no adequate communication in German, French, or English was possible as well as cases where the families had returned to their home country. In the fully participating group, too, the percentage of foreign nationals was quite high, exceeding the proportion of 36% in the Zurich CPT population. This is surprising, because it contradicts previous results in maltreatment research. For example, Finkelhor et al. [14] reported significantly higher attrition rates for ethnic minorities in a follow-up of a nationally representative sample of maltreated children in the United States. The higher participation rate of foreign nationals in our sample may be due partly to the fact that the authority of medical institutions may be seen as higher by the migrant population than by Swiss citizens [15]. As many families had moved at follow-up, an enormous effort was put into finding new addresses. Contrary to our expectation, moves were not overrepresented in non-participants. Although moves may indicate problems, they do not necessarily decrease participation in child maltreatment research if a new address is available. Unexpectedly, no variable associated with time-related and logistic barriers – school age of child, single parenthood, large distances – was more common in non-participants than in participants. Although not tested for statistical significance due to low numbers, the rate of refusals was quite high in children placed out-of-home. We suppose that external placement is an indicator of highly dysfunctional families [16,17]. For these children, we usually contacted the child welfare professionals looking after the child, who in turn asked the parents for permission to participate or referred us directly to the parents. Those parents mostly refused participation, however.

Besides the demographic variables, maltreatment characteristics were tested for unevenness in distribution in participants and non-participants. However, of the characteristics associated with high intrusive quality, neither substantiated maltreatment nor sexual abuse nor intrafamilial perpetrator was more common in non-participants. The latter result is surprising, as other studies at our hospital with a highly traumatized population where traumas had not been inflicted by caregivers had much higher participation rates than this study [18-22]. Although they are not part of the family, the extrafamilial perpetrators were usually known to the family and close to the child (e.g., sports coaches). Therefore, the confrontation with extrafamilial maltreatment may still be perceived as more intrusive than with traumas following severe traffic accidents, for example.

There are certain limitations inherent in these analyses of characteristics for recruitment bias in a maltreatment outcome study. First, the variables presented represent only a small selection of the factors that may be associated with participation. Other possibly correlated variables of great interest, such as socioeconomic status, psychiatric disorders of parents, or disciplinary practices [8], were not analyzed, as they were unavailable in non-participants. The lack of socioeconomic status is especially regrettable, as this factor may be associated with foreign nationality [cf. [1]]. There was a possible hint of economic difficulties in the non-contactable group in that many mobile phones answered with the recorded phrase "the number you have dialed is currently not in service," which is often due to unpaid mobile phone bills.

Still further variables may have influenced participation. Although the voluntary nature of participation was emphasized in the information letter and telephone call, there is still a chance that some participants did not adequately understand this or doubted the fact that non-participation would have no influence on future treatments. Participants may also have been the people who were more satisfied with the hospital intervention in their case. Still other participants may have had a hidden agenda: For example, one mother went to look for the hospital cleaning team after the interview in order to apply for a job; where the children had difficulties in school performance, some parents hoped to receive an expert's report on the results of the developmental examination showing that the child has satisfactory cognitive abilities. Second, the population seen by the CPT at University Children's Hospital Zurich is not fully representative of maltreated children and may differ in severity or frequency of different types of maltreatment. Third, cases in which no contact or adequate communication in German, French, or English could be established were grouped together, because there was neither refusal nor consent to participate. However, it is possible that reasons for not participating differed within this group. Finally, although we were able to offer communication in the two most common languages in Switzerland, German and French, and in addition in English, the leading language of international discourse, it should be noted that Switzerland hosts important minority groups speaking Serbo-Croatian, Albanian, Portuguese, or Turkish, some members of which we were unable to reach.

## Conclusions

The current study is one of the few to give an account of possible biases in recruiting a sample of maltreated children for an outcome study. Barriers to participation in maltreatment studies are high, and future research should be concerned with factors that improve the participation rate. Participation may be higher if, unlike in this study, the institution conducting the follow-up is independent of the institution to which the child was originally referred.

The results have implications for the procedure of maltreatment research. As non-contacts were partly due to inability to adequately communicate in German, French, or English, highly skilled interviewers with different cultural backgrounds should be used to include more different nationalities. Positive findings are that moves and logistic barriers were not significantly associated with non-participation. Therefore, not only researchers but also clinical professionals are encouraged to spare no effort in finding the new addresses of maltreated children's families, because once found they are as likely to participate as non-movers.

Further research is needed to support these results in different national backgrounds and to test for biases in variables not included here, especially socioeconomic status. This will entail gathering more detailed information on non-participants, while respecting ethical boundaries. Overall, the fact that only the child's nationality was unevenly distributed between participants and non-participants is encouraging.

## Competing interests

The authors declare that they have no competing interests.

## Authors' contributions

All authors participated equally in the study design. AJ collected the data, performed the statistical analyses, and drafted the manuscript. UL and ML revised the manuscript. All authors read and approved the final manuscript.
